# Parenchymal loop technique using a novel 0.018-inch guidewire in endoscopic ultrasound-guided pancreatic duct drainage

**DOI:** 10.1055/a-2740-3836

**Published:** 2025-11-28

**Authors:** Ritsuko Oishi, Haruo Miwa, Kazuki Endo, Hiromi Tsuchiya, Yuichi Suzuki, Manabu Morimoto, Shin Maeda

**Affiliations:** 1 26437 Gastroenterological Center, Yokohama City University Medical Center, Yokohama, Japan; 2 Department of Gastroenterology, Yokohama City University Graduate School of Medicine, Yokohama, Japan

Endoscopic ultrasound-guided pancreatic duct drainage (EUS-PDD) is indicated when transpapillary pancreatic duct drainage is unsuccessful 1 2 . EUS-PDD with a 22-gauge needle is effective for non-dilated pancreatic ducts; however, it is limited to a 0.018-inch guidewire. Because of its small caliber, a 0.018-inch guidewire easily migrates outside of the pancreatic duct through the puncture tract, making the procedure difficult and causing guidewire shearing 3 4 . A novel 0.018-inch guidewire (J-wire premier NM; J-Mit, Kyoto, Japan) features a highly flexible and extended tip, which facilitates loop formation. In addition, the absence of visible markers reduces resistance within the needle, allowing safe withdrawal ( Fig. 1 ). These characteristics enable what we term the “parenchymal loop technique”. In this approach, the guidewire, even after migration into the parenchyma, can be advanced into the pancreatic duct by forming a loop ( Fig. 2 and Video 1 ).

**Fig. 1:**
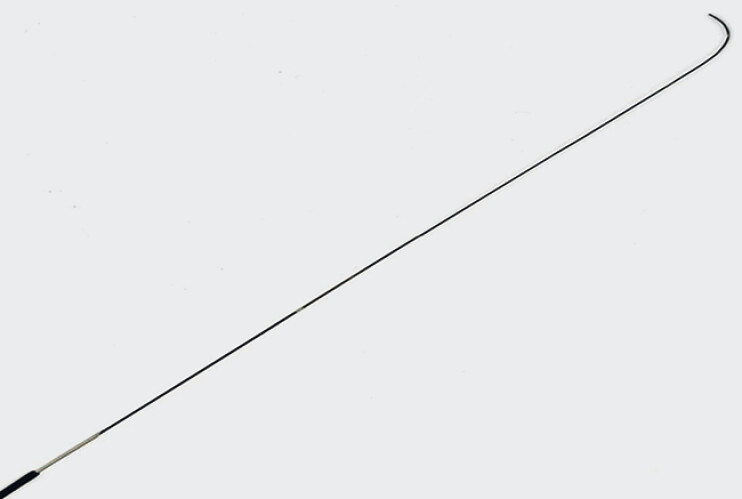
A novel 0.018-inch guidewire (J-wire premier NM; J-Mit, Kyoto, Japan) features a highly flexible and extended tip, which facilitates loop formation. In addition, the absence of visible markers reduces resistance within the needle, allowing safe withdrawal.

**Fig. 2:**
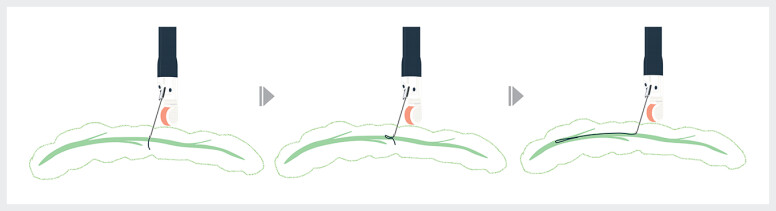
Schema of the “parenchymal loop technique”. When the novel 0.018-inch guidewire tip migrates into the pancreatic parenchyma, gentle further advancement pushes it back and inverts it, leading to loop formation and enabling successful insertion into the main pancreatic duct.

A novel 0.018-inch guidewire enabled endoscopic ultrasound-guided pancreatic duct drainage using the parenchymal loop technique.Video 1

A 63-year-old man with chronic pancreatitis was admitted to our hospital with symptomatic pancreatic duct stones. Because the transpapillary approach was difficult owing to duodenal edema, EUS-PDD was performed ( Fig. 3 ). First, the main pancreatic duct, measuring only 1.1 mm on EUS, was punctured with a 22-gauge needle. After puncture, the contrast medium was injected, and the novel 0.018-inch guidewire was introduced. Although the guidewire repeatedly migrated into pancreatic parenchyma, it could be withdrawn without stacking. When the tip migrated into the parenchyma, gentle further advancement caused it to be pushed back and inverted, leading to loop formation and enabling successful insertion into the main pancreatic duct ( Fig. 4 ). The tract was dilated with a drill dilator, and the 0.018-inch guidewire was exchanged for a 0.025-inch guidewire. Finally, a 7-Fr plastic stent (Through&Pass Type IT; Gadelius Medical, Tokyo, Japan) was successfully deployed.

**Fig. 3:**
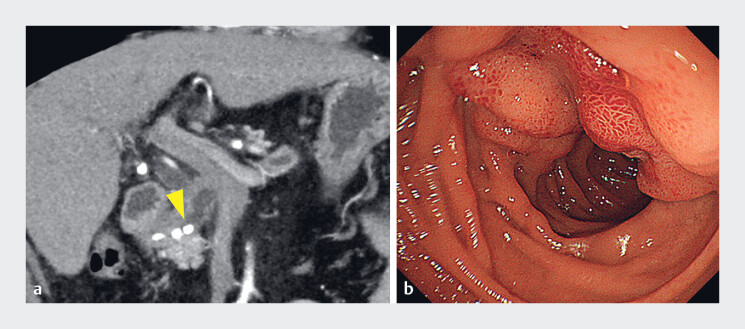
**a** A computed tomography image shows pancreatic duct stones causing obstruction of the main pancreatic duct (arrowhead). **b** An endoscopic image demonstrates duodenal edema, making the transpapillary approach difficult.

**Fig. 4:**
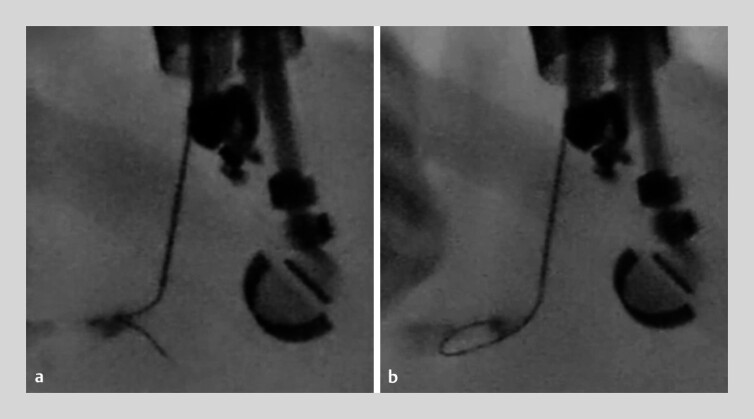
Fluoroscopic images of the parenchymal loop technique. **a** Although the novel 0.018-inch guidewire repeatedly migrates into the pancreatic parenchyma, it can be withdrawn safely without shearing. **b** When the guidewire tip migrates into the pancreatic parenchyma, gentle further advancement pushes it back and inverts it, leading to loop formation and enabling successful insertion into the main pancreatic duct.

To the best of our knowledge, this is the first report of EUS-PDD achieved by the parenchymal loop technique using a novel 0.018-inch guidewire.

Endoscopy_UCTN_Code_TTT_1AS_2AD

## Notes

Endoscopy E-Videos https://eref.thieme.de/e-videos
*E-Videos* is an open access online section of the journal *Endoscopy* , reporting on interesting cases and new techniques in gastroenterological endoscopy. All papers include a high-quality video and are published with a Creative Commons CC-BY license. Endoscopy E-Videos qualify for HINARI discounts and waivers and eligibility is automatically checked during the submission process. We grant 100% waivers to articles whose corresponding authors are based in Group A countries and 50% waivers to those who are based in Group B countries as classified by Research4Life (see: https://www.research4life.org/access/eligibility/ ). This section has its own submission website at https://mc.manuscriptcentral.com/e-videos .
